# Expression Patterns and Functional Analysis of Three *SmTAT* Genes Encoding Tyrosine Aminotransferases in *Salvia miltiorrhiza*

**DOI:** 10.3390/ijms242115575

**Published:** 2023-10-25

**Authors:** Shuai Dong, Long Wang, Huiting Qin, Hongbin Zhan, Donghao Wang, Xiaoyan Cao

**Affiliations:** Key Laboratory of the Ministry of Education for Medicinal Resources and Natural Pharmaceutical Chemistry, College of Life Science, Shaanxi Normal University, Xi’an 710062, China; dong_shuai@snnu.edu.cn (S.D.); sxd2021wl@snnu.edu.cn (L.W.); qinhuiting@snnu.edu.cn (H.Q.); zhanhongbin20213134@snnu.edu.cn (H.Z.); wangdonghao@snnu.edu.cn (D.W.)

**Keywords:** tyrosine aminotransferase, *Salvia miltiorrhiza*, catalytic activity, transient over-expression

## Abstract

Tyrosine aminotransferase (TAT, E.C. 2.6.1.5) is a pyridoxal phosphate-dependent aminotransferase that is widely found in living organisms. It catalyzes the transfer of the amino group on tyrosine to α-ketoglutarate to produce 4-hydroxyphenylpyruvic acid (4-HPP) and is the first enzyme for tyrosine degradation. Three *SmTATs* have been identified in the genome of *Salvia miltiorrhiza* (a model medicinal plant), but their information is very limited. Here, the expression profiles of the three *SmTAT* genes (*SmTAT1*, *SmTAT2*, and *SmTAT3*) were studied. All three genes expressed in different tissues and responded to methyl jasmonate stimuli. *Sm*TAT proteins are localized in the cytoplasm. The recombinant *Sm*TATs were subjected to in vitro biochemical properties. All three recombinant enzymes had TAT activities and *Sm*TAT1 had the highest catalytic activity for tyrosine, followed by *Sm*TAT3. Also, *Sm*TAT1 preferred the direction of tyrosine deamination to 4-HPP, while *Sm*TAT2 preferred transamination of 4-HPP to tyrosine. In parallel, transient overexpression of *SmTATs* in tobacco leaves revealed that all three *Sm*TAT proteins catalyzed tyrosine to 4-HPP in vivo, with *Sm*TAT1 exhibiting the highest enzymatic activity. Overall, our results lay a foundation for the production of tyrosine-derived secondary metabolites via metabolic engineering or synthetic biology in the future.

## 1. Introduction

*Salvia miltiorrhiza* Bunge is a well-known medicinal plants in the family Lamiaceae, and its dried root has used for the treatment of chest pains and cardiovascular diseases [1]. Its main active ingredients include fat-soluble tanshinones and water-soluble phenolic acids [2]. Phenolic acids, especially rosmarinic acid (RA) and salvianolic acid B (SalB), have strong scavenging ability for free radicals and hypolipidemic, anti-inflammatory, and analgesic effects [3]. Identifying genes involved in the biosynthesis and regulation of phenolic acids in *S. miltiorrhiza* has attracted increasing attention recently [4]. As far as we know, 29 genes involved in the biosynthesis of SalB have been identified in the *S. miltiorrhiza* genome, including three *SmTATs* [5].

Tyrosine aminotransferase (TAT, E.C. 2.6.1.5), the first enzyme involved in the metabolic degradation of tyrosine, is a pyridoxal phosphate-dependent aminotransferase that is widely distributed in living organisms. It is essential for the survival and viability of organism [6]. The enzyme catalyzes the reversible transamination between tyrosine and 4-hydroxyphenylpyruvate (4-HPP) [7]. In plants, 4-HPP serves as a precursor for the biosynthesis of various tyrosine-derived plant natural products such as tocopherols, RA, plastoquinone, cyanogenic glycosides, and benzylisoquinoline alkaloids [8,9,10].

In most microbes, 4-HPP is the intermediate of the tyrosine biosynthetic pathway, and TATs are usually responsible for the final step of tyrosine biosynthesis from 4-HPP [11]. However, TAT activity has been proposed to be involved in tyrosine catabolism by deamination rather than tyrosine synthesis in most plants [12]. The broad substrate specificity of TAT allows it to use glutamate and phenylalanine as amino group donors and p-hydroxy-phenylpyruvate, phenylpyruvate, and alpha-ketocaproic acid as amino group acceptors [13]. The catalytic condition of the enzyme is mild and the catalytic efficiency is high without regeneration of the coenzyme cycle [14].

The biochemical and structural properties of TATs from microbes and mammals have been understood in considerable detail [13]. However, the characterization and function of TAT in plants has not been well studied. Genes encoding TATs belong to a small multigene family. There are eight putative *TAT* genes in the *Arabidopsis* genome [12], four TAT genes in *Malus domestica* [11], and three TATs in *S. miltiorrhiza* [5]. In *Arabidopsis*, *At*TAT1 had a higher activity towards tyrosine compared with *At*TAT2. *At*TAT1 and *At*TAT2 have both distinct and shared functions in tyrosine metabolism and *At*TAT1 plays a major role in tocopherol biosynthesis compared to *At*TAT2 [15].

TAT plays an important role in plant growth and development. TAT gene is responds to multiple abiotic stresses and is induced by methyl jasmonate (MeJA) [16]. Drought and low nitrogen stress increase TAT activity in poplar roots, thereby affecting RA content [17]. Most recent studies show that *At*TAT1 is involved in tyrosine catabolism and contributes to plant survival in the dark [18]. *Md*TAT2 overexpression increases resistance to drought and osmotic stress in *Malus domestica* [11].

In this study, three *SmTAT* genes in *S. miltiorrhiza* were cloned and their expression patterns were analyzed. The recombinant proteins of the three *Sm*TATs were subjected to in vitro catalytic reactions and their substrate specificity was compared. Furthermore, their catalytic activities toward tyrosine were confirmed in vivo by transient over-expression system in the leaves of *Nicotiana benthamiana*. To our knowledge, this is the first study about biochemical characterization of TAT in *S. miltiorrhiza*. The results provide valuable information for the production of tyrosine-derived secondary metabolites, such as SalB and RA, via metabolic engineering in the future.

## 2. Results

### 2.1. Bioinformatics Analysis of SmTATs in S. miltiorrhiza

**Figure 1:**
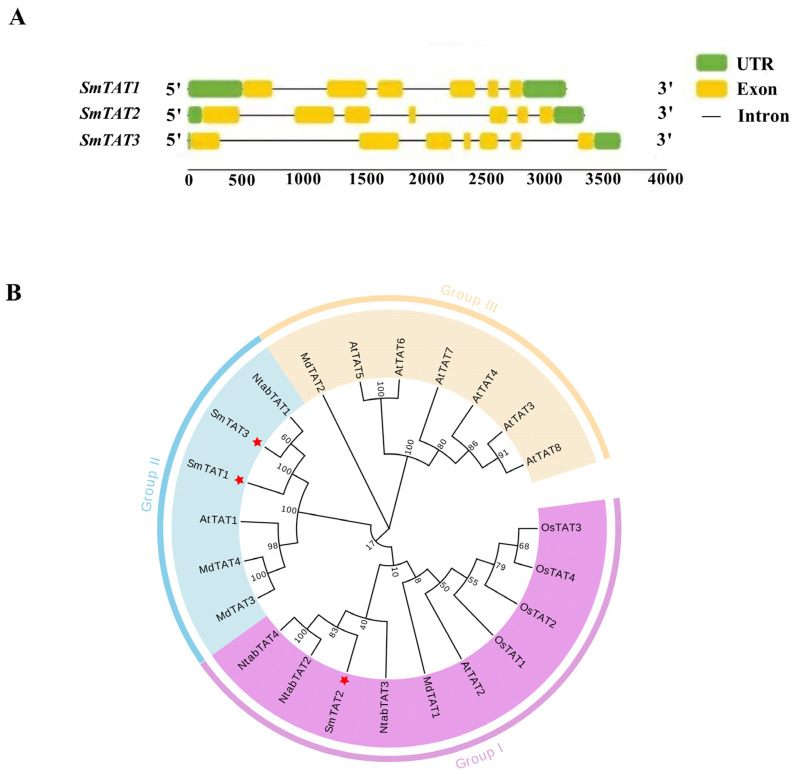
Bioinformatics analysis of *SmTATs*. (**A**) Gene structures of *SmTATs*. (**B**) Phylogenetic evolutionary tree of the TATs from *Arabidopsis thaliana*, *Nicotiana benthamiana*, *Salvia miltiorrhiza*, *Oryza sativa*, and *Malus domestica*.

Gene structure analysis of the three *SmTATs* was shown in Figure 1A. There are 5 introns in the *SmTAT1*, and 6 in *SmTAT2* and *SmTAT3.* The ORF of *SmTAT1*, *SmTAT2*, and *SmTAT3* was 1236 bp, 1308 bp, and 1260 bp, respectively. Amino acid sequences of the three *Sm*TATs and TATs from *Arabidopsis*, *N. benthamiana*, *Oryza sativa*, and *Malus domestica* were used to create a phylogenetic tree. As shown in Figure 1B, all the TATs were clustered into three groups. *Sm*TAT1, *Sm*TAT3 and *At*TAT1 (At5g53970) were clustered in group II, while *Sm*TAT2 and *At*TAT2 (At5g36160) were clustered together. The promoter sequences of *SmTATs* were analyzed using the PlantCare database. *Cis*-elements such as ABRE (abscisic acid response element) and some light response elements were found in the promoter regions of all the three *SmTATs* (Supplementary Table S1), suggesting that *SmTATs* may respond to phytohormone and environmental stress during the growth and development of *S. miltiorrhiza.*

### 2.2. Expression Patterns of SmTATs

**Figure 2:**
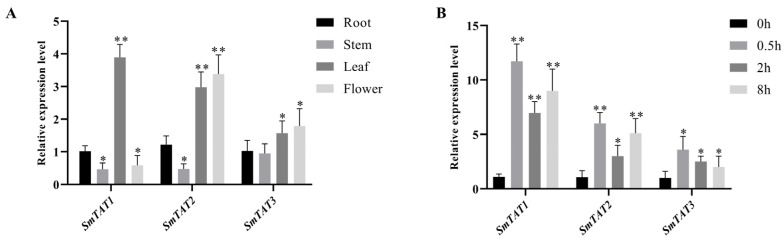
Expression profiles of *SmTATs* in *S. miltiorrhiza*. (**A**) Transcription levels of *SmTATs* in different tissues. (**B**) Expression changes in *SmTATs* in response to MeJA treatments. All data are the means of three biological replicates, with error bars indicating SD. One-way ANOVA (followed by Tukey’s comparisons) tested for significant differences between means (indicated by different letters at *p*  <  0.05). * and ** represent a significant difference at *p*  <  0.05 and *p*  <  0.01 levels, respectively, compared with the control.

The qRT-PCR results showed that *SmTAT1*, *SmTAT2*, and *SmTAT3* were expressed in different tissues, including roots, stems, leaves, and flowers. And all the three members had higher transcription levels in the leaves (Figure 2A). In addition, the expression levels of all the three *SmTATs* were significantly increased under the treatment of MeJA (Figure 2B).

**Figure 3:**
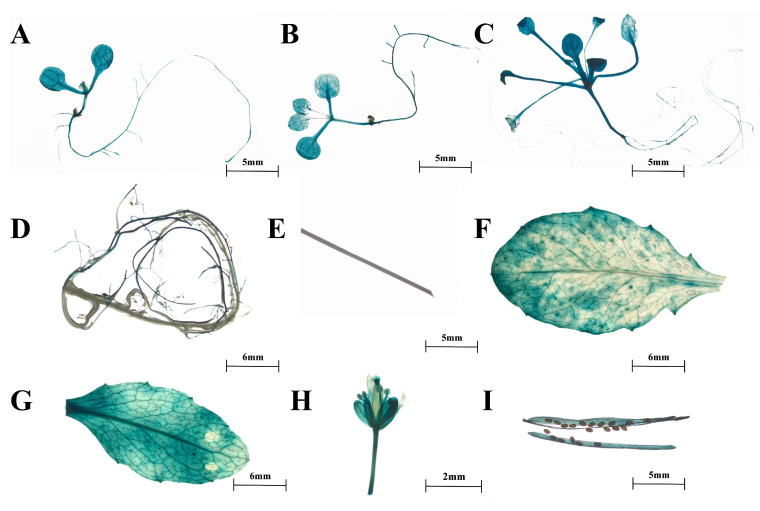
β-glucuronidase (GUS) staining results of *Arabidopsis* expressing *proSmTAT1::GUS*. (**A**) Bud stage; (**B**) four-leaf stage; (**C**) eight-leaf stage; (**D**–**G**) root (**D**), stem (**E**), rosette leaf (**F**), and stem leaf (**G**) at the flowing stage; (**H**) flower; (**I**) silique.

**Figure 4:**
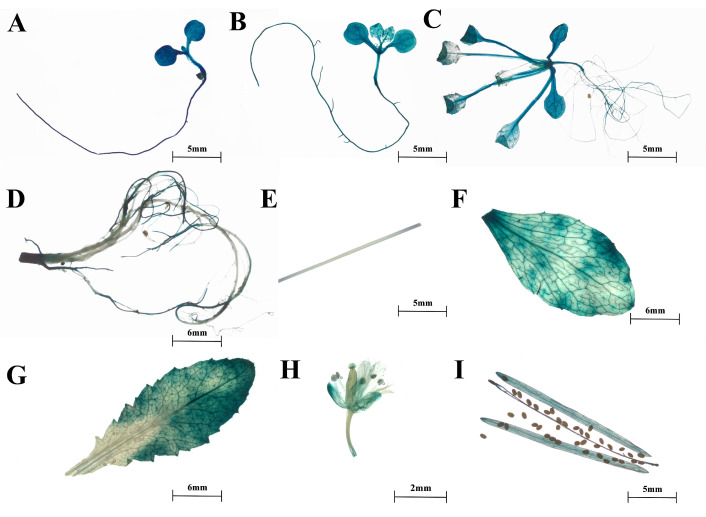
β-glucuronidase (GUS) staining results of *Arabidopsis* expressing *proSmTAT2::GUS*. (**A**) Bud stage; (**B**) four-leaf stage; (**C**) eight-leaf stage; (**D**–**G**) root (**D**), stem (**E**), stem leaf (**F**), and rosette leaf (**G**) at the flowing stage; (**H**) flower; (**I**) silique.

**Figure 5:**
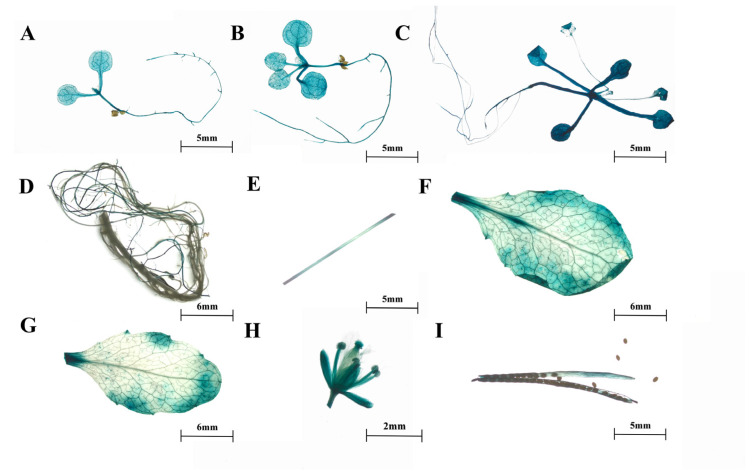
β-glucuronidase (GUS) staining results of *Arabidopsis* expressing *proSmTAT3::GUS*. (**A**) Bud stage; (**B**) four-leaf stage; (**C**) six-leaf stage; (**D**–**G**) root (**D**), stem (**E**), stem leaf (**F**), and rosette leaf (**G**) at the flowing stage; (**H**) flower; (**I**) silique.

Furthermore, we obtained transgenic *Arabidopsis* expressing *proSmTATs::GUS* to investigate the spatiotemporal expression patterns of the three *SmTATs*. GUS staining indicated that the spatiotemporal expression patterns of the three *SmTATs* were basically consistent, and GUS signals could be observed at different developmental stages (Figure 3, Figure 4 and Figure 5). Strong GUS signals were obvious in all the young seedlings of *Arabidopsis* expressing *GUS* driven by *proSmTAT1*, *proSmTAT2*, or *proSmTAT3*. At the flowering stage, the GUS expression profiles in the root was consistent for *Arabidopsis* expressing *proSmTATs::GUS*, with obvious signal in the lateral root, while no GUS signal was observed in the taproot; The GUS signal in the stem leaf was stronger than that in the rosette leaf for *Arabidopsis* expressing *proSmTAT1::GUS*; the GUS signal in the flower driven by *proSmTAT2* was comparatively weaker, while the signal in the stem driven by *proSmTAT3* was comparatively stronger.

### 2.3. SmTATs Are Located in the Cytoplasm

**Figure 6:**
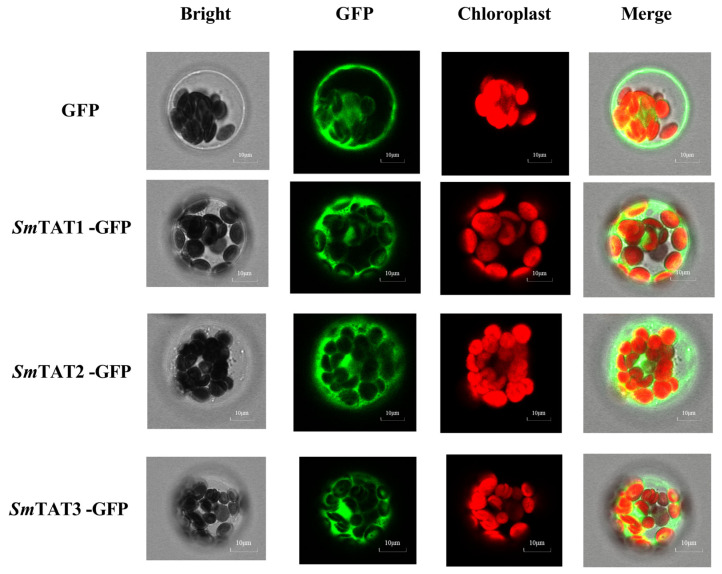
Subcellular location of *Sm*TAT1, *Sm*TAT2, and *Sm*TAT3 in *Arabidopsis* protoplasts.

To determine the subcellular localization of the three *Sm*TAT proteins, we created the fusion expression vector *35Spro::SmTATs-eGFP*. The fusion expression vector was transferred into *Arabidopsis* protoplasts, with *35Spro::eGFP* serving as a positive control. As shown in Figure 6, the green fluorescence of GFP was uniformly distributed throughout the cells, whereas the green fluorescence of the *Sm*TAT1/2/3-GFP fusion proteins was specifically localized in the cytoplasm. Our results indicated that *Sm*TAT1/2/3 are cytoplasmic proteins.

### 2.4. Prokaryotic Expression and Enzyme Assays of SmTATs

**Figure 7:**
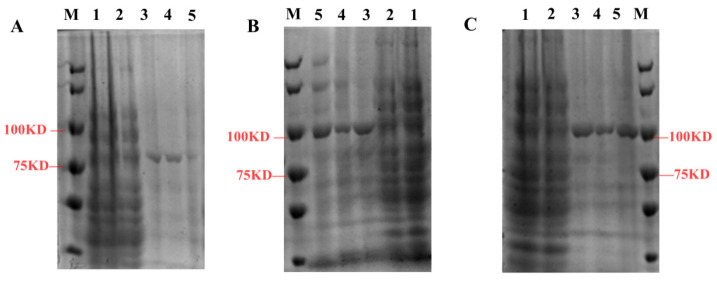
Purification of the recombinant fusion proteins GST-*Sm*TAT1 (**A**), His-*Sm*TAT2 (**B**), and His-*Sm*TAT3 (**C**) by affinity chromatography. M: protein marker; lanes 1–2: proteins in *E. coli* containing pGEX-4T-1 or pET28a-MBP. Lanes 3–5: recombinant proteins.

To analyze the enzyme activities of *Sm*TATs, the recombinant proteins were expressed in *E. coli* and purified by affinity chromatography column. The recombinant fusion proteins GST-*Sm*TAT1, His-*Sm*TAT2, and His-*Sm*TAT3 were analyzed by SDS-PAGE (Figure 7).

**Figure 8:**
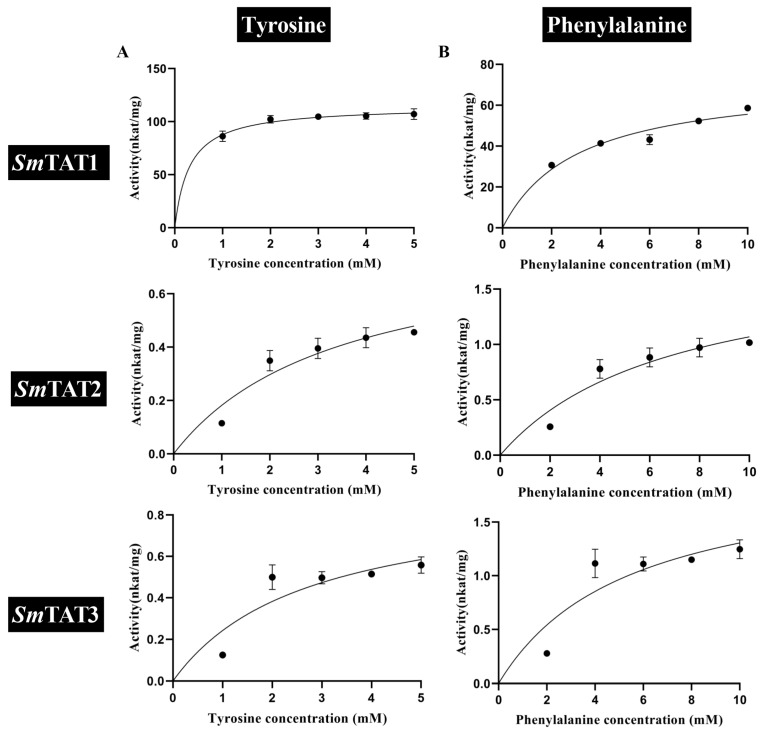
Steady-state kinetic analysis of *Sm*TATs using various amino donors with α-ketoglutarate keto acceptor. Purified recombinant *Sm*TATs were incubated at 30 °C for 30 min with 100 μM PLP, 10 mM α-ketoglutarate, and indicated concentrations of an amino donor. Data are means ± SE (*n* ≥ 3). (**A**) Tyrosine (Tyr) with 1 μg/mL TAT1 or 20 μg/mL TAT2 and TAT3, (**B**) phenylalanine (Phe) with 1 μg/mL TAT1 or 20 μg/mL TAT2 and TAT3. K_m_ and V_max_ are shown in Table 1.

When α-ketoglutarate was used as the amino acceptor, the affinity of *Sm*TAT1 for tyrosine (K_m_ of 0.31 mM) was higher than that of *Sm*TAT2 and *Sm*TAT3 (Table 1, Figure 8A). The catalytic efficiency of *Sm*TAT1 for tyrosine was 1240 and 744 times higher than that of *Sm*TAT2 and *Sm*TAT3, respectively. When phenylalanine was used as the amino donor, the affinity of *Sm*TAT1 for phenylalanine (K_m_ of 3.08 mM) was higher than that of *Sm*TAT2 and *Sm*TAT3 (K_m_ of 6.93 and 5.47 mM) (Table 1, Figure 8B), and the catalytic efficiency of *Sm*TAT1 for phenylalanine were 6.88 and 39 times higher than that of *Sm*TAT2 and *Sm*TAT3, respectively.

**Figure 9:**
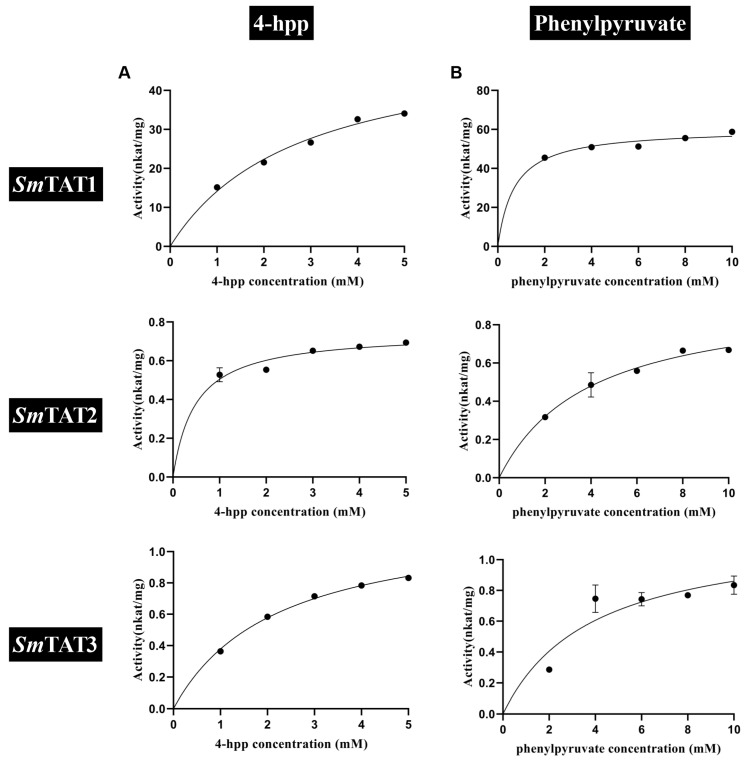
Steady-state kinetic analysis of *Sm*TATs using various keto acceptors with Glu amino donor. Purified recombinant *Sm*TATs were incubated at 30 °C for 30 min with 100 μM PLP, 10 mM Glu, and indicated concentrations of a keto acceptor. Data are means ± SE (*n* ≥ 3). (**A**) 4-hydroxyphenylpyruvate (4-hpp) with 1 μg/mL TAT1 or 20 μg/mL TAT2 and TAT3, (**B**) phenylpyruvate (ppy) with 1 μg/mL TAT1 or 20 μg/mL TAT2 and TAT3. K_m_ and V_max_ are shown in Table 1.

In the reverse reaction, when glutamate was used as the amino donor, the affinity of *Sm*TAT2 for 4-HPP was highest (K_m_ of 0.48 mM) (Table 1, Figure 9A), and the catalytic efficiency of *Sm*TAT2 for 4-HPP was 0.92 and 3.33 times higher than that of *Sm*TAT1 and *Sm*TAT3, respectively. The affinity of *Sm*TAT1 for phenylpyruvate was higher (K_m_ of 0.71 mM) than that of *Sm*TAT2 and *Sm*TAT3 (Table 1, Figure 9B). The catalytic efficiency of *Sm*TAT2 for phenylpyruvate was 326 and 203 times higher than that of *Sm*TAT2 and *Sm*TAT3, respectively.

Overall, the catalytic efficiency of *Sm*TAT1 was higher than *Sm*TAT2 and *Sm*TAT3. *Sm*TAT1 showed the highest substrate affinity for tyrosine and preferred to transfer the amino group on tyrosine to α-ketoglutarate, while *Sm*TAT2 preferred the reverse reaction to convert 4-HPP to tyrosine.

**Figure 10:**
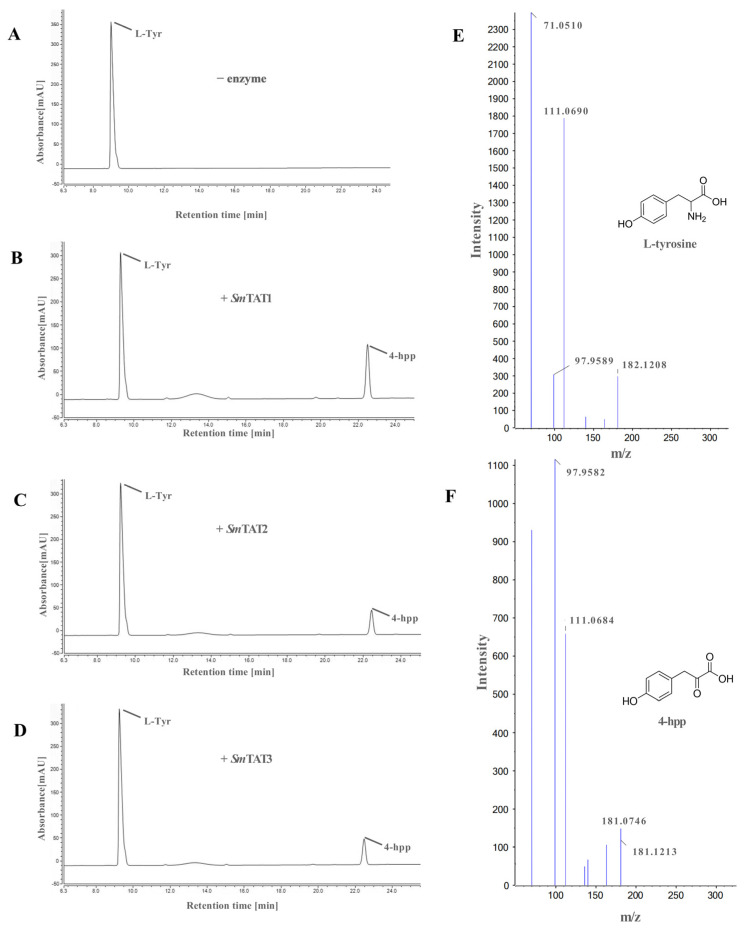
Tyrosine aminotransferase activity of the recombinant *Sm*TATs. (**A**–**D**) HPLC profiles of the enzymatic reaction after incubation of no enzyme, *Sm*TAT1, *Sm*TAT2, and *Sm*TAT3 for 30 min, with α-ketoglutarate as the amino acceptor. (**E**,**F**) Mass spectrum and structure of tyrosine (Tyr) and 4-hydroxyphenylpyruvate (4-HPP).

The catalytic activities of recombinant *Sm*TATs to transfer the amino group on tyrosine to α-ketoglutarate in vitro were confirmed by HPLC. As shown in chromatogram (Figure 10), all the three *Sm*TATs had tyrosine aminotransferase activity and *Sm*TAT1 had the highest catalytic efficiency for tyrosine transamination to 4-HPP. UPLC-QTOF-MS was used to identify the reaction product 4-HPP.

### 2.5. Transient Overexpression of SmTATs Increased the Content of 4-HPP in the Leaves of N. benthamiana

**Figure 11:**
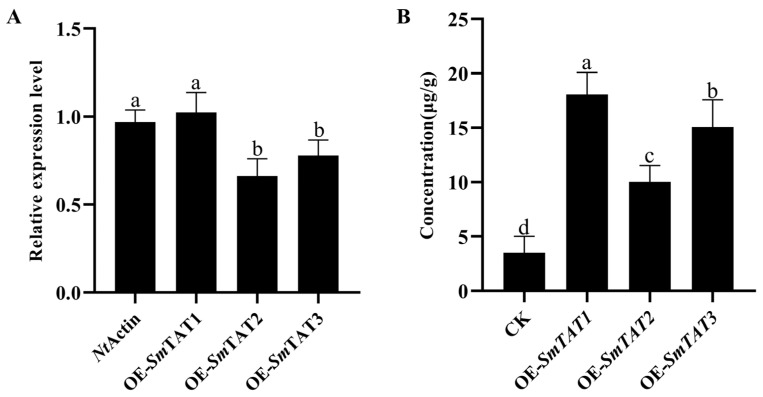
Transient over-expression of *SmTAT1/2/3* in *N. benthamiana* leaves. (**A**) Transcription levels of *SmTATs* by qRT-PCR. (**B**) Determination of 4-HPP by UPLC-QTOF-MS. All data are the means of three biological replicates, with error bars indicating SD. One-way ANOVA (followed by Tukey’s comparisons) tested for significant differences between means. Different letters indicate significant difference between the two samples at the level of *p* < 0.05.

To further verify the enzyme activity of *Sm*TATs in vivo, we transiently overexpressed *SmTATs* in the leaves of *N. benthamiana*. Our qRT-PCR showed that a high transcription level of *SmTAT1*/*2*/*3* was detected in the transgenic tobacco leaves (Figure 11A). Meanwhile, we determined the content of 4-HPP by UPLC-QTOF-MS. The results showed that the content of pHPL was significantly higher in *SmTAT1*/*2*/*3-*overexpressed leaves than the control (Figure 11B). The content of 4-HPP in the control group was 3.5 μg/g, whereas the content of 4-HPP in the leaves overexpressing *SmTAT1*, *SmTAT2*, and *SmTAT3* were 18.24 μg/g, 10.11 μg/g, and 15.19 μg/g, respectively. The tyrosine aminotransferase activity of the three *Sm*TATs was confirmed in *N. benthamiana*.

## 3. Discussion

Since the publication of the genome sequence of *S. miltiorrhiza*, a well-known medicinal plant, there has been an increasing number of studies on the function of genes involved in the biosynthesis and regulation of active ingredients [19]. Three genes encoding TAT have been identified in *S. miltiorrhiza* [5], but their information is very limited. In the present study, we systematically compared the expression patterns of the three genes and their biochemical characteristics were also investigated. The results lay a foundation for the production of tyrosine-derived nature compounds by metabolic engineering or synthetic biology.

Our qRT-PCR results showed that all the three *SmTATs* were highly expressed at different tissues of the two-year-old *S. miltiorrhiza*, with a higher level in the leaves. The TAT-mediated tyrosine metabolic pathway generates plastoquinone and ubiquinone, which function as electron and proton carriers in the electron transport chain of photosynthesis and respiration [20]. We speculate that *SmTAT1*, *SmTAT2*, and *SmTAT3* may play a role in photosynthesis and respiration. *SmTAT1* showed the highest transcription level in the stems of two-month-old *S. miltiorrhiza* plantlet [21]. We found that the expression level was lowest in the stem of two-year-old plant (Figure 2A). The promoter sequence determines the expression pattern of a gene [22]. In this study, we obtained *proSmTATs::GUS* transgenic *Arabidopsis* and performed GUS staining. The GUS signals were basically consistent with the qRT-PCR results.

Jasmonates are signal molecules of defense/stress pathways and MeJA is an effective elicitor to enhance the production of phenolic acids in *S. miltiorrhiza* [23]. Previous study indicated that the transcription levels of all the three *SmTATs* in the hairy roots of *S. miltiorrhiza* were significantly up-regulated under the treatment of MeJA [5], which are consistent with our results (Figure 2B). TAT functions in abiotic stress environment. For instance, three *MdTATs* in apple were induced by drought stress and confer tolerance to drought and osmotic stresses in plants [11]. Whether *SmTATs* are responsive to drought stress in *S. miltiorrhiza* deserves further investigation.

Prokaryotic expression system has been widely used to obtain large amounts of recombinant proteins [24]. However, the recombinant proteins expressed by prokaryotic cells are expressed too fast, resulting in aggregation of the expressed protein molecules into inactive inclusion bodies [25]. To obtain recombinant *Sm*TATs, three *E. coli* expression systems (pGEX-4T-1, pET-28a, and pET-28a-MBP) were used in this study. Soluble *Sm*TAT1 was successfully obtained by the prokaryotic expression vector pGEX-4T-1. The recombinant *Sm*TAT2 and *Sm*TAT3 tended to be expressed in the form of inclusion bodies. We finally obtain bioactive *Sm*TAT2 and *Sm*TAT3 from inclusion bodies expressed in pET-28a-MBP via direct dilution.

TAT catalyzes the reversible transamination between tyrosine and 4-HPP [7]. TATs are responsible for the tyrosine biosynthesis from 4-HPP in most microbes [11], while TAT is involved in tyrosine catabolism by deamination rather than tyrosine synthesis in most plants [12]. TAT can also use glutamate and phenylalanine as amino group donors and p-hydroxy-phenylpyruvate, phenylpyruvate, and alpha-ketocaproic acid as amino group acceptors [13]. *Pv*TAT from *Prunella vulgaris* had substrates preference for tyrosine and favored the conversion of tyrosine to 4-HPP [26]. In *Arabidopsis*, *At*TAT1 and *At*TAT2 have a distinct substrate specificity and preferred reaction direction. *At*TAT1 had a higher activity towards tyrosine than *At*TAT2. Also, *At*TAT1 favored the direction of tyrosine deamination to 4-HPP, whereas *At*TAT2 preferred the reverse reaction [12]. Here, we found that the catalytic efficiency of *Sm*TAT1 is higher than *Sm*TAT2 and *Sm*TAT3. *Sm*TAT1 showed the highest substrate affinity for tyrosine and preferred the transfer of amino group on tyrosine to α-ketoglutarate, while *Sm*TAT2 preferred the reverse reaction to convert 4-HPP to tyrosine (Table 1 and Figure 8). Interesting, as shown in Figure 1, *At*TAT1 and *Sm*TAT1 (two members preferred the conversion of tyrosine to 4-HPP) are clustered in the same group, while *At*TAT2 and *Sm*TAT2 (preferred the reaction of 4-HPP to tyrosine) are clustered in another group (Figure 1). The biochemical characterization of the TATs support the phylogenetic evolutionary tree. It should be mentioned that the low concentrations of substrate are missing in kinetic analysis because of shortcomings of the experimental design. In future studies, we will overcome the shortcoming to obtain more accurate kinetic parameters.

TAT is a key enzyme in the biosynthesis of tyrosine-derived secondary metabolites in plants, such as phenolic acids, uronic acids, tocopherols, and alkaloids [27]. The overexpression of *PvTAT* in *Prunella vulgaris* hairy roots increased the content of RA [26]. Virus-induced silencing of *TAT* led to the reduction in isoquinoline alkaloid content in *opium poppy* [8]. In the hairy roots of *S. miltiorrhiza*, overexpressing *SmTAT1* alone did not significantly increase RA content, but the co-expression of *SmTAT1* and *SmHPPR1* resulted in a 4.3 times increase in RA content [28]. For the two homologous TAT proteins in *Arabidopsis*, *At*TAT1 plays a major role in tocopherol biosynthesis compared to *At*TAT2 [15]. In the present study, in vitro and in vivo experiments supported that *Sm*TAT1 exhibited higher activity in converting tyrosine to 4-HPP. Many MeJA responsive transcription factors, such as *Sm*MYC2 [29], *Sm*MYB97 [30], and *Sm*MYB111 [31], regulate the production of phenolic acids in *S. miltiorrhiza* by binding to the promoter of *SmTAT1* and activate its expression. We speculated that *Sm*TAT1 plays a dominant role in the biosynthesis of phenolic acids among the three homologous TAT proteins in *S. miltiorrhiza.*

In conclusion, three *SmTAT* genes in *S. miltiorrhiza* were cloned and their spatiotemporal expression profiles were comparatively analyzed. All the three genes significantly responded to MeJA stimuli and the *Sm*TAT proteins are all located in the cytoplasm. In vitro catalytic reactions of the recombinant *Sm*TATs indicated that all the three *Sm*TATs had tyrosine aminotransferase activity and *Sm*TAT1 had the highest catalytic efficiency for tyrosine transamination to 4-HPP. Transient overexpression of *SmTATs* in tobacco leaves indicated that all three *Sm*TAT proteins have catalytic activities toward tyrosine. To further confirm their role to secondary metabolites, we will overexpress and knockout *SmTAT* genes in *S. miltiorrhiza* to analyze whether they affect the biosynthesis of rosmarinic acid in the future.

## 4. Materials and Methods

### 4.1. Plant Materials and Growth Conditions

Two-year-old *Salvia miltiorrhiza* in the experimental field of Shaanxi Normal University was collected at the flowering stage and the roots, stems, leaves, and flowers were separately frozen in liquid nitrogen for RNA extraction. Two-month-old *S. miltiorrhiza* plantlets were used for the treatment of methyl jasmonate (MeJA) as we described previously [32].

*Arabidopsis thaliana* ecotype Columbia-0 and *Nicotiana benthamiana* seedlings were cultured in a culture room at 23 °C and a humidity of 55% under 16 h of light and 8 h of darkness. While *Arabidopsis* used for subcellular localization experiment was cultured under short light condition (8 h light, 16 h dark).

### 4.2. Isolation of SmTAT1/2/3 and Sequence Analysis

Total RNA was extracted from two-month-old *S. miltiorrhiza* using the Plant Total RNA Extraction Kit (Vazyme, Nanjing, China) and then reverse transcribed into cDNA using Hiscript II reverse transcriptase (Vazyme, Nanjing, China). Genomic DNA was extracted from two-month-old *S. miltiorrhiza* using the DN15-Plant DNA Mini Kit (Aidlab, Beijing, China). The open reading frames (ORFs) of *SmTAT1*, *SmTAT2*, and *SmTAT3* were amplified with *SmTATs*-F/R primers (Supplementary Table S2). The promoter regions of *SmTAT1*, *SmTAT2*, and *SmTAT3*, which is 1350 bp, 1125 bp, and 1495 bp, respectively, were amplified with *ProSmTATs*-F/R primers (Supplementary Table S2). The amplified fragments were inserted into the vector pMD19-T (TaKaRa, Dalian, China) and confirmed by sequencing.

A phylogenetic evolutionary tree of TATs from different species was created using MEGA X software (1000 bootstraps) by the neighbor-joining method, and then embellished by the online EvolView tool (http://www.evolgenius.info/evolview, accessed on 3 June 2023). The *cis*-elements in the promoter regions were predicted using the PlantCare online website (http://bioinformatics.psb.ugent.be/webtools/plantcare/html/, accessed on 5 December 2022).

All primers used in this study are listed in Supplementary Table S2.

### 4.3. Expression Analysis via qRT-PCR

Total RNA was extracted from different tissues of *S. miltiorrhiza* and seedlings treated with MeJA and then reverse transcribed to cDNA. Real-time quantitative PCR (qRT-PCR) was performed on a Roche LightCycler 96 system (Roche Diagnostics GmbH, Basel, Switzerland) using SYBR Green qPCR mixture (Vazyme, Nanjing, China). *SmUbiquitin* was used as internal reference gene. Expression levels of *SmTAT1/2/3* were analyzed via 2^−∆∆CT^ analysis [33].

### 4.4. Expression and Purification of SmTATs from Escherichia coli

The ORF of *SmTAT1* was inserted into the *Eco*R I and *Not* I sites of the pGEX-4T-1 vector. The obtained vector pGEX-4T-1-*SmTAT1* was transformed into Arctic Express (DE3) strain for protein expression. The transgenic Arctic Express strain was cultured, harvested, and lysed by ultrasound. The recombinant *Sm*TAT1 was isolated and purified according to the previous method [34].

The ORFs of *SmTAT2* and *SmTAT3* were, respectively, inserted into the *Sal* I\*Xho* I sites and the *Bam*H I\*Sal* I sites of the modified pET28a which carries a MBP tag (pET28a-MBP). The recombinant pET28a-MBP-*SmTAT2* and pET28a-MBP-*SmTAT3* were transformed into BL21 (DE3) strain for protein expression, respectively. The transgenic BL21 strain was cultured in 100 mL LB medium with 100 mg/L kanamycin at 37 °C until OD_600_ = 0.3–0.4, and then isopropyl-β-d-thiogalactoside was added with the final concentration of 1mM to induce the expression of *SmTAT2* and *SmTAT3* at 16 °C until OD_600_ = 0.8. The cell cultures were harvested by centrifugation and lysed with 30 mL buffer A (500 mM Tric-HCL, 0.15 mM NaCl, 5 mM EDTA, 1 mg/mL lysozyme, pH = 8). The lysed cells were subjected to centrifugation at 12,000× *g* for 15 min at 4 °C. The resulting pellets were washed with 20 mL buffer B (500 mM Tric-HCl, 0.15 mM NaCl, 2.5 M urea, 5 mM EDTA, pH = 8), and purified using the NI-NTA column (QualitYard, Beijing, China). The purified proteins were washed from the column with 2 mL 8 M urea, then denatured overnight at 4 °C with 40 mL buffer D (500 mM Tric-HCl, 0.15 mM NaCl, 5 mM EDTA, pH = 8).

The recombinant proteins were identified via SDS-PAGE.

### 4.5. In Vitro Enzyme Assays

The in vitro aminotransferase reaction of the recombinant *Sm*TAT1/2/3 was performed as described previously by Prabhu [35]. The increased products including 4-HPP, phenylpyruvate, tyrosine, and phenylalanine were detected at 331 nm, 320 nm, 280 nm, and 260 nm, respectively, by a Synergy H4 Hybrid Multi-Mode Microplate Reader (Bio Tek, Winooski, VT, USA). The catalytic activity to transfer the amino group on tyrosine to α-ketoglutarate was confirmed by an UltiMate 3000 HPLC System (Thermo Fisher Scientific, Waltham, MA, USA) equipped with a ZORBAX SB-C18 column (4.6 × 250 mm, 5 µm; Agilent Technologies, Santa Clara, CA, USA). The mobile phase was acetonitrile (A) and 0.1% trifluoroacetic acid (B). The solvent gradient was as follows: 0–25 min: A 10–55%; 25–27 min: A 55–100%; 27–30 min: A 100%; 30–30 min: A 100–10%. The standards of tyrosine, α-ketoglutarate, phenylpyruvate, phenylalanine, glutamate, and 4-HPP were purchased from Solarbio (Beijing, China).

### 4.6. Agrobacterium-Mediated Transient Expression in N. benthamiana

According to the Gateway manufacturer’s protocol (Invitrogen, Carlsbad, CA, USA), the ORF of *SmTAT1/2/3* was constructed into the pDNOR-207 vector via BP reaction to generate the entry vector pDONR207*-SmTAT1/2/3*. Then, *SmTATs* were recombined into the pEarleyGate 202 vector to obtain the overexpression vectors pEarleyGate202-*SmTATs*.

Agroinfltration of *N. benthamiana* leaves with *Rhizobium radiobacter* strain EHA105, carrying either pEarleyGate202-*SmTATs* or pEarleyGate202 empty vector, was performed according to the previous protocols [36]. The infiltrated leaves were cultured in the dark for 48 h and then collected for qRT-PCR to detect the expression levels of *SmTATs*. *NtActin* was used as the reference gene.

### 4.7. UPLC-QTOF/MS Analysis

To determine the concentration of 4-HPP, the infiltrated leaves were subjected to extraction according to the previous protocol [37] and detected by ultra-performance liquid chromatography quadrupole time-of-flight mass spectrometry (UPLC-QTOF-MS) (AB SCIEX Triple TOF 5600plus, Framingham, Framingham, MA, USA) in a positive ionization mode. We performed the UPLC at the following conditions: the mobile phase comprised 0.1% trifluoroacetic acid (A) and acetonitrile (B). The solvent gradient was as follows: 0–5 min, 10–25% B; 5–6 min, 25% B; 6–10 min, 25–55% B; 10–12 min, 55% B; 12–13 min, 55–100% B; 13–14 min, 100–10% B. The flow rate was 0.3 mL/min and the injection volume was 2.0 μL. The detection wavelengths were 331 nm.

The conditions for MS analysis were as follows: The MSE scanning range was *m*/*z* 80−800, and the scanning time was 0.2 s. Ion source was set at the temperature of 120 °C, capillary voltage of 1.0 kV, cone voltage of 40 V, desolvent temperature of 400 °C, and collision voltage of 20 eV (4-HPP, t_R_ = 0.562 min, *m*/*z* 181.14 [M-H]^+^).

### 4.8. Heterologous Expression of proSmHPPRs::GUS and GUS Staining

The promoter regions of *SmTAT1*, *SmTAT2*, *and SmTAT3* were, respectively, inserted into the pCAMBIA1391Z via double digestion to drive the expression of *GUS*. The *Arabidopsis* expressing *ProSmHPPRs::GUS* was acquired through the inflorescence dip method [38]. The T_2_ generation transgenic lines at different development stages were used for GUS staining as previously described [39].

### 4.9. Subcellular Localization of SmTAT Proteins

The ORFs of *SmTAT1/2/3* without stop codon were inserted into pHBT-GFP-NOS vector via double digestion. The recombinant plasmids pHBT-GFP-NOS-*SmTAT1/2/3* were transferred into *Arabidopsis* protoplasts as described previously [40]. The transformed protoplasts were grown at 21 °C for 14 h under the dark. Then, the fluorescence signal was observed and photographed under confocal laser microscopy (Leica TCS SP5, LEICA, Wetzlar, Germany) to confirm the subcellular localization of *Sm*TATs.

### 4.10. Statistic Analysis

Two statistical significance between different groups was analyzed by SPSS 20.0 using Student’s *t*-test. Differences with *p* < 0.05 were considered significant. Data were expressed as means ± SD.

## Figures and Tables

**Table 1:** Kinetic parameters of *Sm*TAT1, *Sm*TAT2, and *Sm*TAT3 enzymes.

Variable-Substrate	Co-Substrate	K_m_ (mM)	V_max_ (nmol s^−1^ mg^−1^)	k_cat_ (s^−1^)	k_cat_/K_m_ (mM^−1^ s^−1^)
*Sm*TAT1					
Tyr	α-KG	0.31 ± 0.11	114.7 ± 5.41	19.83 ± 0.93	74.4 ± 29.4
Phe	α-KG	3.08 ± 0.99	72.69 ± 8.31	12.57 ± 1.43	4.68 ± 2.02
4-HPP	Glu	2.77 ± 0.52	53.2 ± 4.61	9.21 ± 0.78	3.5 ± 0.94
PPY	Glu	0.71 ± 0.21	60.46 ± 2.48	10.45 ± 0.39	16.31 ± 4.62
*Sm*TAT2					
Tyr	α-KG	3.47 ± 1.69	0.81 ± 0.21	0.15 ± 0.03	0.06 ± 0.04
Phe	α-KG	6.93 ± 3.39	1.81 ± 0.47	0.33 ± 0.08	0.68 ± 0.24
4-HPP	Glu	0.48 ± 0.16	7.47 ± 0.45	1.35 ± 0.08	3.23 ± 1.24
PPY	Glu	3.94 ± 0.99	0.95 ± 0.11	0.17 ± 0.02	0.05 ± 0.02
*Sm*TAT3					
Tyr	α-KG	2.73 ± 1.61	0.90 ± 0.26	0.16 ± 0.04	0.1 ± 0.08
Phe	α-KG	5.47 ± 3.23	2.02 ± 0.59	0.35 ± 0.11	0.12 ± 0.09
4-HPP	Glu	2.22 ± 0.22	12.17 ± 0.51	2.12 ± 0.09	0.97 ± 0.14
PPY	Glu	3.91 ± 2.01	1.21 ± 0.26	0.21 ± 0.05	0.08 ± 0.05

α-KG, α-ketoglutarate; 4-HPP, 4-hydroxyphenylpyruvate; PPY, phenylpyruvate; Tyr, tyrosine; Phe, phenylalanine; Glu, glutamate. Data are means ± SE (*n* ≥ 3).

## Data Availability

Data will be made available on request.

## Supplementary Materials

The following supporting information can be downloaded at: https://www.mdpi.com/article/10.3390/ijms242115575/s1.

## References

[1] Jung I., Kim H., Moon S., Lee H., Kim B. (2020). Overview of *Salvia miltiorrhiza* as a potential therapeutic agent for various diseases: An update on efficacy and mechanisms of action. Antioxidants.

[2] Zhou L., Zuo Z., Chow M.S. (2005). Danshen: An overview of its chemistry, pharmacology, pharmacokinetics, and clinical use. J. Clin. Pharmacol..

[3] Cao W., Guo X.W., Zheng H.Z., Li D.P., Jia G.B., Wang J. (2012). Current progress of research on pharmacologic actions of salvianolic acid B. Chin. J. Integr. Med..

[4] Zhao Z., Yun C., Gu L., Liu J., Yao L., Wang W., Wang H. (2023). Melatonin enhances biomass, phenolic accumulation, and bioactivities of rosemary (*Rosmarinus officinalis*) in vitro shoots under UV-B stress. Physiol. Plant.

[5] Wang B., Sun W., Li Q., Li Y., Luo H., Song J., Sun C., Qian J., Zhu Y., Hayward A. (2015). Genome-wide identification of phenolic acid biosynthetic genes in *Salvia miltiorrhiza*. Planta.

[6] Sasidharan S., Saudagar P. (2022). Knockout of tyrosine aminotransferase gene by homologous recombination arrests growth and disrupts redox homeostasis in *Leishmania Parasite*. Parasitol. Res..

[7] Mizukami H., Ellis B.E. (1991). Rosmarinic acid formation and differential expression of tyrosine aminotransferase isoforms in *Anchusa officinalis* cell suspension cultures. Plant Cell Rep..

[8] Lee E.J., Facchini P.J. (2011). Tyrosine aminotransferase contributes to benzylisoquinoline alkaloid biosynthesis in opium poppy. Plant Physiol..

[9] Zhang S., Yan Y., Wang B. (2014). Selective responses of enzymes in the two parallel pathways of rosmarinic acid biosynthetic pathway to elicitors in *Salvia miltiorrhiza* hairy root cultures. J. Biosci. Bioeng..

[10] Kim Y.B., Kim J.K., Uddin M.R., Xu H., Park W.T., Tuan P.A., Li X., Chung E., Lee J.H., Park S.U. (2013). Metabolomics analysis and biosynthesis of rosmarinic acid in *Agastache rugosa* Kuntze treated with methyl jasmonate. PLoS ONE.

[11] Wang H., Dong Q., Duan D., Zhao S., Li M., Van Nocker S., Ma F., Mao K. (2018). Comprehensive genomic analysis of the TYROSINE AMINOTRANSFERASE (TAT) genes in apple (*Malus domestica*) allows the identification of MdTAT2 conferring tolerance to drought and osmotic stresses in plants. Plant Physiol. Biochem..

[12] Wang M., Toda K., Maeda H.A. (2016). Biochemical properties and subcellular localization of tyrosine aminotransferases in *Arabidopsis thaliana*. Phytochemistry.

[13] Mehere P., Han Q., Lemkul J.A., Vavricka C.J., Robinson H., Bevan D.R., Li J. (2010). Tyrosine aminotransferase: Biochemical and structural properties and molecular dynamics simulations. Protein Cell.

[14] Koper K., Hataya S., Hall A.G., Takasuka T.E., Maeda H.A. (2023). Biochemical characterization of plant aromatic aminotransferases. Methods Enzymol..

[15] Wang M., Toda K., Block A., Maeda H.A. (2019). TAT1 and TAT2 tyrosine aminotransferases have both distinct and shared functions in tyrosine metabolism and degradation in *Arabidopsis thaliana*. J. Biol. Chem..

[16] Antognoni F., Faudale M., Poli F., Biondi S. (2009). Methyl jasmonate differentially affects tocopherol content and tyrosine amino transferase activity in cultured cells of *Amaranthus caudatus* and *Chenopodium quinoa*. Plant Biol..

[17] Zhang C., Meng S., Li M., Zhao Z. (2018). Transcriptomic insight into nitrogen uptake and metabolism of *Populus simonii* in response to drought and low nitrogen stresses. Tree Physiol..

[18] Zhu F., Alseekh S., Koper K., Tong H., Nikoloski Z., Naake T., Liu H., Yan J., Brotman Y., Wen W. (2022). Genome-wide association of the metabolic shifts underpinning dark-induced senescence in *Arabidopsis*. Plant Cell.

[19] Zhou C., Lin C., Xing P., Li X., Song Z. (2022). SmGDB: Genome database of *Salvia miltiorrhiza*, an important TCM Plant. Genes Genom..

[20] Liu L., Yang D., Liang T., Zhang H., He Z., Liang Z. (2016). Phosphate starvation promoted the accumulation of phenolic acids by inducing the key enzyme genes in *Salvia miltiorrhiza* hairy roots. Plant Cell Rep..

[21] Huang B., Yi B., Duan Y., Sun L., Yu X., Guo J., Chen W. (2008). Characterization and expression profiling of tyrosine aminotransferase gene from *Salvia miltiorrhiza* (Dan-shen) in rosmarinic acid biosynthesis pathway. Mol. Biol. Rep..

[22] Habener J.F. (1985). Regulation of polypeptide-hormone biosynthesis at the level of the genome. Am. J. Physiol..

[23] Ge Q., Zhang Y., Hua W.P., Wu Y.C., Jin X.X., Song S.H., Wang Z.Z. (2015). Combination of transcriptomic and metabolomic analyses reveals a JAZ repressor in the jasmonate signaling pathway of *Salvia miltiorrhiza*. Sci. Rep..

[24] Hussain H., Fisher D.I., Abbott W.M., Roth R.G., Dickson A.J. (2017). Use of a protein engineering strategy to overcome limitations in the production of “Difficult to Express” recombinant proteins. Biotechnol. Bioeng..

[25] Jäger V.D., Kloss R., Grünberger A., Seide S., Hahn D., Karmainski T., Piqueray M., Embruch J., Longerich S., Mackfeld U. (2019). Tailoring the properties of (catalytically)-active inclusion bodies. Microb. Cell Factories.

[26] Ru M., Wang K., Bai Z., Peng L., He S., Wang Y., Liang Z. (2017). A tyrosine aminotransferase involved in rosmarinic acid biosynthesis in *Prunella vulgaris* L. Sci. Rep..

[27] Rippert P., Puyaubert J., Grisollet D., Derrier L., Matringe M. (2009). Tyrosine and phenylalanine are synthesized within the plastids in *Arabidopsis*. Plant Physiol..

[28] Xiao Y., Zhang L., Gao S., Saechao S., Di P., Chen J., Chen W. (2011). The c4h, tat, hppr and hppd genes prompted engineering of rosmarinic acid biosynthetic pathway in *Salvia miltiorrhiza* hairy root cultures. PLoS ONE.

[29] Du T., Niu J., Su J., Li S., Guo X., Li L., Cao X., Kang J. (2018). SmbHLH37 functions antagonistically with SmMYC2 in regulating jasmonate-mediated biosynthesis of phenolic acids in *Salvia miltiorrhiza*. Plant Sci..

[30] Li L., Wang D., Zhou L., Yu X., Yan X., Zhang Q., Li B., Liu Y., Zhou W., Cao X. (2020). JA-responsive transcription factor SmMYB97 promotes phenolic acid and tanshinone accumulation in *Salvia miltiorrhiza*. J. Agric. Food Chem..

[31] Yang R., Li S., Dong S., Wang L., Qin H., Zhan H., Wang D., Cao X., Xu H. (2023). SmJAZ4 interacts with SmMYB111 or SmMYC2 to inhibit the synthesis of phenolic acids in *Salvia miltiorrhiza*. Plant Sci..

[32] Peng J.J., Wu Y.C., Wang S.Q., Niu J.F., Cao X.Y. (2020). SmbHLH53 is relevant to jasmonate signaling and plays dual roles in regulating the genes for enzymes in the pathway for salvianolic acid B biosynthesis in *Salvia miltiorrhiza*. Gene.

[33] De Vandesompele J., Preter K., Pattyn F., Poppe B., Van Roy N., De Paepe A., Speleman F. (2022). Accurate normalization of real-time quantitative RT-PCR data by geometric averaging of multiple internal control genes. Genome Biol..

[34] Yang Q.Q., Hua W.P., Zou H.L., Yang J.X., Wang X.Z., Zhang T., Wang D.H., Zhu X.J., Cao X.Y. (2022). Overexpression of SmLAC25 promotes lignin accumulation and decreases salvianolic acid content in *Salvia miltiorrhiza*. Plant Sci..

[35] Prabhu P.R., Hudson A.O. (2010). Identification and partial characterization of an L-tyrosine aminotransferase (TAT) from *Arabidopsis thaliana*. Biochem. Res. Int..

[36] Sparkes I.A., Runions J., Kearns A., Hawes C. (2006). Rapid, transient expression of fluorescent fusion proteins in tobacco plants and generation of stably transformed plants. Nat. Protoc..

[37] Dong S., Kong G., Qutob D., Yu X., Tang J., Kang J., Dai T., Wang H., Gijzen M., Wang Y. (2012). The NLP toxin family in *Phytophthora sojae* includes rapidly evolving groups that lack necrosis-inducing activity. Mol. Plant Microbe Interact..

[38] Clough S.J., Bent A.F. (1998). Floral dip: A simplified method for Agrobacterium-mediated transformation of *Arabidopsis thaliana*. Plant J. Cell Mol. Biol..

[39] Jefferson R.A., Kavanagh T.A., Bevan M.W. (1987). GUS fusions: Beta-glucuronidase as a sensitive and versatile gene fusion marker in higher plants. EMBO J..

[40] Gomes-Junior R.A., Moldes C.A., Delite F.S., Pompeu G.B., Gratão P.L., Mazzafera P., Lea P.J., Azevedo R.A. (2006). Antioxidant metabolism of coffee cell suspension cultures in response to cadmium. Chemosphere.

