# Supplementary material for: Expression Patterns and Functional Analysis of Three SmTAT Genes Encoding Tyrosine Aminotransferases in Salvia miltiorrhiza
Source: Int J Mol Sci. 2023 Oct 25;24(21):15575. doi: 10.3390/ijms242115575 (PMC10649420; doi:10.3390/ijms242115575)
Supplement: Supplementary file 1 [file ijms-24-15575-s001.zip › Table S1 Cis-Elements of SmTATs.pdf]

Table S1.

Cis-acting elements analysis of *SmTAT1* promoter

Cis-Elements	Sequence	Number	Functions
ABRE	ACGTG	1	abscisic acid responsiveness element
LTR	CCGAAA	1	involved in low-temperature responsiveness
TATC-box	TATCCCA	1	involved in gibberellin-responsiveness
G-box	CACGTT	2	involved in light responsiveness
ABRE	ACGTG	2	involved in the abscisic acid responsiveness
MBS	CAACTG	3	involved in drought-inducibility

Cis-acting elements analysis of *SmTAT2* promoter

Cis-Elements	Sequence	Number	Functions
ABRE	ACGTG	3	abscisic acid responsiveness element
TGA-element	AACGAC	1	involved in auxin-responsive element
ARE	AAACCA	2	related to the anaerobic induction
G-box	CACGTC	3	involved in light responsiveness
WUN-motif	AAATTTCCT	1	related to the wound-responsive element
circadian	CAAAGATATC	1	involved in circadian control

Cis-acting elements analysis of *SmTAT3* promoter

Cis-Elements	Sequence	Number	Functions
ABRE	ACGTG	4	abscisic acid responsiveness element
TGACG-motif	TGACG	2	involved in the MeJA-responsiveness
ABRE	ACGTG	4	abscisic acid responsiveness element
G-box	TACGTG	2	involved in light responsiveness
MBS	CAACTG	2	involved in drought-inducibility
P-box	CCTTTTG	3	gibberellin-responsive element
