# Supplementary material for: Expression Patterns and Functional Analysis of Three SmTAT Genes Encoding Tyrosine Aminotransferases in Salvia miltiorrhiza
Source: Int J Mol Sci. 2023 Oct 25;24(21):15575. doi: 10.3390/ijms242115575 (PMC10649420; doi:10.3390/ijms242115575)
Supplement: Supplementary file 1 [file ijms-24-15575-s001.zip › Table S2 Primers.pdf]

Table S2. List of primers used in the study.

Primer	Oligo Sequence 5′ to 3′
RT-*SmUbiquitin*-F	ACCCTCACGGGGAAGACCATC
RT-*SmUbiquitin*-R	ACCACGGAGACGGAGGACAAG
RT-*NtActin-F*	AGTAAGCAACTGGGACGATA
RT-*NtActin-R*	CCACTAAGGACGATGTTTCC
SmTAT1--F	ATGGAGTTGCAGAATCCAGC
SmTAT1--R	TTAGTAGGAATGCCGTTCACAG
SmTAT2--F	ATGCCAAATTCTACTGAGAGAGAG
SmTAT2--R	TCATTGTTTCTTGGCGTGC
SmTAT2--F	ATGGATGTCCGAAACAATGGT
SmTAT2--R	CTATATTTCTATTGCTTGGGGAGG
RT-*SmTAT1*-F	CAACTGCTGGTCTTCCACAAAC
RT-*SmTAT1*-R	GCGAGCCAAAACGGACA
RT-*SmTAT2*-F	CGGAGATCCATCCGCCTTCC
RT-*SmTAT2*-R	CGGGAATACCGACGGTGGAG
RT-*SmTAT3*-F	TGCTGAAACTGCCAAGAGGCT
RT-*SmTAT3*-R	CCGGGCACCAACCATCTCTT
proSmTAT1-F	TACGGAGCCGCCCCTAAAAA
proSmTAT1-R	TGCCGCAACTGAATCGAGAG
proSmTAT2-F	ATAAGTTGTTTGGGTAGCC
proSmTAT2-R	TGTAGCGAAGGAGGAAAG
proSmTAT3-F	GGGACGACACAAAGTTTT
proSmTAT3-R	AGCATCAGTTGCGTGCTG
1391Z-*proSmTAT1*-F	CCCAAGCTTTACGGAGCCGCCCCTAAAAA
1391Z-*proSmTAT1*-R	CGGGATCCTGCCGCAACTGAATCGAGAG
1391Z-*proSmTAT2*-F	CCCAAGCTTATAAGTTGTTTGGGTAGCC
1391Z-*proSmTAT2*-R	CGGGATCCTGTAGCGAAGGAGGAAAG
1391Z-*proSmTAT3*-F	CCCAAGCTTGGGACGACACAAAGTTTT
1391Z-*proSmTAT3*-R	CGGGATCCAGCATCAGTTGCGTGCTG
pGEX-*SmTAT1*--F	GGAATTCATGGAGTTGCAGAATCCAGC
pGEX-*SmTAT1*--R	CCGCTCGAGGTAGGAATGCCGTTCACAG
pTEV-*SmTAT2*--F	GCGTCGACAAATGCCAAATTCTACTGAGAGAGAG
pTEV-*SmTAT2*--R	CCGCTCGAGTTTGTTTCTTGGCGTGC
pTEV-*SmTAT3*--F	CGGGATCCATGGATGTCCGAAACAATGGT
pTEV-*SmTAT3*--R	GCGTCGACGTATTTCTATTGCTTGGGGAGG
